# Defects in energy metabolism increase the susceptibility of *Staphylococcus aureus* and its small colony variants (SCVs) to *Staphylococcus lugdunensis* and lugdunin

**DOI:** 10.1128/spectrum.01006-25

**Published:** 2025-08-22

**Authors:** Chao-Chin Liu, Shih-Cheng Chang, Jwu-Ching Shu, Mei-Hui Lin

**Affiliations:** 1 Department of Medical Biotechnology and Laboratory Science, College of Medicine, Chang Gung University 71589, Taoyuan, Taiwan; 2 Graduate Institute of Biomedical Sciences, College of Medicine, Chang Gung University 210836, Taoyuan, Taiwan; 3 Department of Medical Laboratory, Chang Gung Memorial Hospital at Linkou 38014, Taoyuan, Taiwan; University of Calgary, Calgary, Alberta, Canada

**Keywords:** *Staphylococcus aureus*, *Staphylococcus lugdunensis*, lugdunin, small colony variant, energy metabolism

## Abstract

Lugdunin, produced by *Staphylococcus lugdunensis*, exhibits potent antimicrobial activity against a broad range of Gram-positive bacteria, including *Staphylococcus aureus*. This study aims to elucidate the mechanisms underlying the susceptibility of *S. aureus* to lugdunin by screening a transposon-based mutant library generated using the lugdunin-susceptible *S. aureus* strain HG001. We identified four *S*. *aureus* mutants with mutations in *ctaA*, *hemY*, *qoxA*, and *guaB*, which are involved in ATP and GTP synthesis, and displayed increased susceptibility to *S. lugdunensis*. Further tests using synthetic lugdunin confirmed that energy generation defects make *S. aureus* more susceptible to lugdunin. Moreover, these mutants displayed small colony variant (SCV) phenotypes, a slow-growing form of *S. aureus* characterized by altered metabolism, revealing the critical role of energy metabolism in *S. aureus* defense mechanisms. Furthermore, we observed that clinical SCV isolates exhibited greater susceptibility to lugdunin compared to their parental strains. Additionally, lugdunin treatment enhanced SCV susceptibility to commonly used antibiotics, including oxacillin and vancomycin, suggesting a synergistic effect between lugdunin and antibiotics. This study highlights the role of lugdunin in sensitizing energy-compromised *S. aureus* to antibiotics, presenting a promising avenue for combating SCV-related infections.

**IMPORTANCE:**

The rise of antimicrobial resistance in *Staphylococcus aureus*, particularly small colony variants (SCVs), poses a significant clinical challenge due to their persistence and reduced susceptibility to antibiotics. *Staphylococcus lugdunensis* produces lugdunin, a potent antimicrobial compound that inhibits *S. aureus* growth. This study reveals that energy metabolism-deficient mutants and clinical SCVs exhibit increased susceptibility to lugdunin, underscoring the role of energy metabolism in bacterial defense. Furthermore, lugdunin enhances SCV susceptibility to antibiotics oxacillin and vancomycin, suggesting a potential strategy to overcome antibiotic resistance. By elucidating the link between energy metabolism and susceptibility to antimicrobial compounds, this study highlights lugdunin as a promising candidate for combating SCV-associated infections and antibiotic-resistant *S. aureus*.

## INTRODUCTION

The increasing resistance of *Staphylococcus aureus* to antimicrobial agents, especially the emergence of methicillin-resistant *S. aureus* (MRSA), presents a major challenge to clinical treatment. These resistant strains limit the effectiveness of first-line antibiotics, complicating the management of infections and leading to increased morbidity and mortality (1, 2). The ability of *S. aureus* to rapidly acquire and disseminate resistance genes further exacerbates the difficulty in controlling its spread, necessitating ongoing research into new antimicrobial agents and treatment strategies (3). Moreover, *S. aureus* can form small colony variants (SCVs), a phenotypic form characterized by slow growth, reduced metabolism, and distinctive colony morphology (4). SCVs commonly arise due to mutations that disrupt the electron transport chain, ATP production, and thymidine synthesis, leading to decreased sensitivity to antibiotics that target actively growing cells (5, 6). As a result, SCVs are frequently associated with chronic, relapsing infections, such as osteomyelitis, endocarditis, and device-related infections (4). Therefore, there is an urgent need to develop new antibiotics and alternative therapeutic strategies to address these challenges.

*S. aureus* and *Staphylococcus lugdunensis* are both commensal bacteria that can coexist within the human nasal cavity. Recent studies have revealed that *S. lugdunensis* produces a unique antimicrobial compound called lugdunin, which exhibits potent activity against a broad spectrum of Gram-positive pathogens, including *S. aureus* (7). Importantly, lugdunin production by *S. lugdunensis* has been associated with eradicating *S. aureus* from the nasal microbiomes of hospitalized patients, underscoring its potential role in modulating nasal microbiota and preventing pathogenic colonization (7–9).

Lugdunin is a cyclic peptide synthesized through a non-ribosomal pathway (7). Its structure comprises a thiazolidine ring and multiple d-amino acids, contributing to its antimicrobial efficacy (10). Lugdunin exerts its inhibitory effect by acting as a cation ionophore, facilitating the influx of cations into bacterial cells and disrupting the membrane potential. This disruption leads to rapid depolarization of the cytoplasmic membrane and acidification of the cytoplasm, ultimately depleting the energy resources of *S. aureus* and inhibiting its growth (11). Lugdunin also forms water-filled channel structures in lipid membranes, compromising bacterial integrity (12). These combined actions effectively inhibit *S. aureus* growth, underscoring the potential of lugdunin as a novel antimicrobial agent. In this study, we investigated the mechanism underlying the susceptibility of *S. aureus* and its SCV derivatives to lugdunin. The results demonstrated that energy generation defects make *S. aureus* and SCVs more susceptible to lugdunin. Treating SCVs with lugdunin enhanced the susceptibility of SCVs to oxacillin and vancomycin. The results suggest a potential therapeutic approach for treating persistent staphylococcal infections.

## MATERIALS AND METHODS

### Bacterial strains and culture conditions

The bacterial strains used in this study are listed in Table 1. Bacteria were cultured in tryptic soy broth (TSB) or agar (TSA) (Oxoid, Basingstoke, UK). A basic medium (BM) was used for the inhibition assay and contained 1% soy peptone, 0.5% yeast extract, 0.5% NaCl, 0.1% glucose, and 0.1% K_2_HPO_4_. 2, 2′- dipyridyl (2-DP) (Sigma-Aldrich) is an iron chelator that creates iron-limited conditions, which have been shown to promote lugdunin biosynthesis in *S. lugdunensis* by activating biosynthetic pathways involved in antimicrobial compound production, including non-ribosomal peptide synthesis such as that of lugdunin (7). Therefore, 2-DP was added to the BM medium to enhance the antimicrobial activity of *S. lugdunensis*.

**TABLE 1:** Bacterial strains used in this study^*a*^

Strains	Properties	Phenotype/antibiotic resistance	Reference or source
*E. coli*			
EPI300	A host for cloning		Epicenter Technologies
*S. aureus*			
RN4220	A restriction-deficient strain derived from *S. aureus* NCTC8325		(13)
HG001	A derivative of *S. aureus* NCTC8325, as wild-type *S. aureus*	MSSA	(13)
M60	*ctaA* mutated HG001	SCV, MSSA	(14)
M60 (pHY-*ctaA*)	Δ*ctaA* with pHY-*ctaA*	MSSA, Tc^r^	(14)
M47	*hemY* mutated HG001	SCV, MSSA	(14)
M47 (pHY-*hemY*)	Δ*hemY* with pHY-*hemY*	MSSA, Tc^r^	(14)
YU1	*qoxA* mutated HG001	SCV, MSSA	This study
YU1 (pGHL-*qoxA*)	Δ*qoxA* with pGHL-*qoxA*	MSSA, Cm^r^	This study
YU2	*guaB* mutated HG001	SCV, MSSA	This study
YU2 (pGHL-*guaB*)	Δ*guaB* with pGHL-*guaB*	MSSA, Cm^r^	This study
LTF1	A clinical strain	MRSA	(15)
LTF22	A clinical strain derived from strain LTF1	SCV, MRSA	(15)
109	A clinical strain	MRSA	(16)
2358B	A clinical strain derived from strain 109	SCV, MRSA	(16)
*S. lugdunensis*			
SL 10	A clinical strain		(17)

^*a*^ SCV: small colony variant. MSSA: methicillin-susceptible *S. aureus*; MRSA: methicillin-resistant *S. aureus*. Tc^r^: tetracycline resistance, Cm^r^: chloramphenicol resistance.

### Inhibition assay

*S. aureus* was first spread on BM plates containing 150 µM 2-DP, followed by inoculating 10 µL of overnight-cultured *S. lugdunensis* L10 onto the plates. To ensure comparable lawn formation in inhibition assays despite differences in growth rate, the inoculum concentrations were adjusted, allowing all strains to achieve full confluence on BM agar plates within 24 h.

After 24 h of incubation, the inhibition zone surrounding the *S. lugdunensis* colony was observed and photographed. The distance from the *S. lugdunensis* colony to the outer edge of the clear inhibition zone was measured in four directions (up, down, left, and right), and the average of these measurements was taken as the size of the inhibition zone. To validate the antimicrobial susceptibility of *S. aureus* strains to lugdunin, the synthetic lugdunin peptide was added to plates that had been spread with *S. aureus*. After incubation, the effect of synthetic lugdunin on the growth of *S. aureus* was observed and photographed. The synthetic lugdunin was purchased from MyBioSource, Inc. (USA) and was reported by the manufacturer to have a purity of ≥95%, as determined by high-performance liquid chromatography. Since the synthetic lugdunin was dissolved in DMSO, a vehicle-only control containing DMSO without lugdunin was included in all experiments using synthetic lugdunin to ensure that the solvent had no effect on bacterial growth.

### Transposon mutagenesis

A mutant library was generated using the *bursa aurealis* transposon according to a method described previously (18). Briefly, *S. aureus* HG001 was sequentially transformed with pBursa and pFA545, which contain a mariner transposable element and genes encoding transposase and selection markers. After inducing transposase expression and curing the plasmids at 43°C, mutants with transposon insertions were isolated. The transposon insertion sites were identified by sequencing.

### Plasmids

The construction of pHY-*ctaA* and pHY-*hemY* was described in the previous study (14). For the complementation of the deletion mutants Δ*qoxA* and Δ*guaB*, the *guaB* gene was amplified with the primers guaB-F (5′-TAAGGATCCCGCAATCTCTGCAATTATTC) and guaB-R (5′-CGCCATATGGTCGTTCTCCTTTATCTTAA); *qoxA* was amplified using the primers qoxA-F (5′-TTCGGATCCCATTTGTAGTATTAGGAGGT) and qoxA-R (5′-ATAGCTAGCTTAATGTCCACCTCCATGAT). The amplified *guaB* DNA fragment was digested with BamHI and NdeI and was then inserted into the BamHI–NdeI sites in pRPO-gfp (19) to generate pGHL-*guaB*. The amplified *qoxA* DNA fragment was digested with BamHI and BmtI and was then inserted into the BamHI–BmtI sites in pRPO-gfp to generate pGHL-*qoxA*.

### ATP assay

The cells were collected, washed, and resuspended in PBS. The cell density was determined spectrophotometrically at 578 nm (A578). Intracellular ATP levels were quantified using the BacTiter-Glo Microbial Cell Viability Assay Kit (Promega, Madison, WI, USA). A 100 µL cell sample was mixed with an equal volume of the assay reagent, and the mixture was incubated at room temperature with shaking for 10 min. Following incubation, the ATP-dependent luminescence was measured using a luminometer (Promega, GloMax Explorer GM-3510). ATP concentration was determined from a standard curve generated with adenosine 5′-triphosphate. The ATP levels were then normalized against the cell density (A578) of the bacterial culture.

### Spot assay

Overnight cultures of *S. aureus* strains were inoculated in fresh TSB to an OD_578_ of 0.05 and subcultured at 37°C with shaking for 4 h. The cell density was then adjusted to an OD_578_ of 1.0 (approximately 1 × 10^8^ CFU/mL) and serial dilutions were prepared. A 10 µL aliquot of each dilution, ranging from 10^1^ to 10^5^ CFU, was spotted on agar plates with varying concentrations of the tested antibiotics. The plates were incubated for 24 h and photographed.

### Statistical analysis

Data were analyzed using a two-tailed Student’s *t*-test in GraphPad Prism software (version 7.0, La Jolla, CA, USA). A *P*-value < 0.05 was considered statistically significant. Results are expressed as mean ± SD.

## RESULTS

### Energy-generation system is involved in *S. aureus* resistance to *S. lugdunensis* inhibition

**Fig 1:**
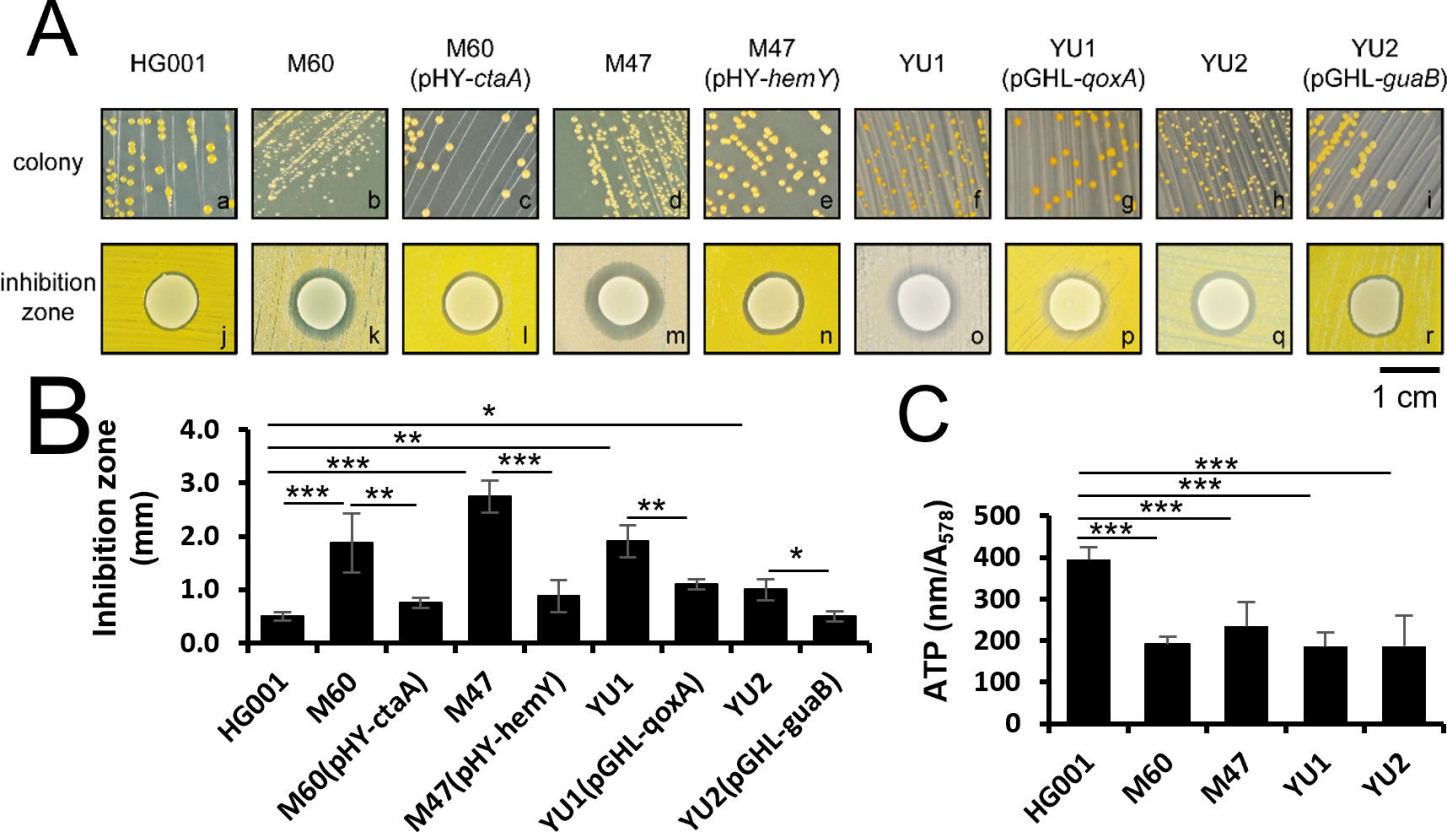
Susceptibility of energy-deficient mutants to *S. lugdunensis* inhibition and their ATP levels. (**A**) BM medium containing 150 µM 2-DP was spread with *S. aureus* HG001, M60, M60(pHY*-ctaA*), M47, M47(pHY-*hemY*), YU1, YU1(pGHL-*qoxA*), YU2, and YU2(pGHL-*guaB*). Ten microliters of overnight-cultured *S. lugdunensis* was then inoculated on the lawns of *S. aureus*. After 24 h of incubation at 37°C, inhibition zones surrounding *S. lugdunensis* colonies were observed and photographed. In order to mitigate the impact of growth rate variation and achieve consistent lawn coverage, inoculum concentrations were adjusted accordingly, allowing both *S. aureus* HG001 and its mutant strains to reach full confluence on BM agar within the 24-h incubation period. (**B**) The inhibition zone size was quantified by measuring the distance from the edge of the *S. lugdunensis* colony to the outermost edge of the clear inhibition zone. (**C**) Intracellular ATP levels of *S. aureus* strains were measured and normalized to *A*_578_ (absorbance at 578 nm). The displayed image is representative of three independent experiments. Data are presented as mean ± SD of triplicate samples. Statistical significance is indicated as *P < 0.05, **P < 0.01, and ***P < 0.001.

A previous study demonstrated that *S. lugdunensis* produces lugdunin to eliminate *S. aureus* (7). To investigate how lugdunin affects *S. aureus*, a mariner-based transposon, *bursa aurealis* (18), was used to mutagenize *S. aureus* HG001. A mutant library, which contains approximately 9,000 mutants, was generated. These transposon-inserted mutants were analyzed for their ability to resist inhibition by *S. lugdunensis*. The inhibitory activity of *S. lugdunensis* was determined by measuring the inhibition zone surrounding the *S. lugdunensis* colony. Following the screening, four mutants, M60 (Δ*ctaA*), M47 (Δ*hemY*), YU1 (Δ*qoxA*), and YU2 (Δ*guaB*) (Table 2), were identified as more susceptible to *S. lugdunensis* inhibition compared to the parental strain *S. aureus* HG001 (Fig. 1A, j, k, m, o, q and B). Susceptibility of these mutants to *S. lugdunensis* inhibition was restored upon plasmid-based complementation (Fig. 1A, l, n, p, r and B). To confirm that the observed phenotypic restoration in the complemented strains reflected actual recovery of gene expression, we measured the mRNA levels of *ctaA*, *hemY*, *qoxA*, and *guaB* by RT-qPCR. Compared to the wild-type strain HG001, transcript levels were significantly reduced in the respective mutants, confirming successful gene disruption. In complemented strains, the expression of the target genes was markedly restored, and in some cases, exceeded wild-type levels, likely due to constitutive expression from the plasmid. These results demonstrated that complementation was effective at both the transcriptional and phenotypic levels (Fig. S2). It is well known that *S. aureus* has deficiencies in heme synthesis and energy metabolism, leading to the development of slow-growing SCVs (20). The Δ*ctaA* and Δ*hemY* mutants, previously reported to have defects in heme synthesis (14), formed small colonies (Fig. 1A, b and d). Similarly, the mutants with defects in *qoxA* and *guaB*, which encode Quinol oxidase subunit II and IMP dehydrogenase, respectively, are involved in ATP and GTP synthesis (21, 22) and also form small colonies (Fig. 1A, f and h). ATP levels analysis revealed that the mutants had lower ATP levels than their parental strain, HG001 (Fig. 1C). These findings suggest that energy synthesis is critical for *S. aureus* in combating *S. lugdunens*is inhibition.

**TABLE 2:** Transposon insertion mutants with increased susceptibility to *S. lugdunens*is inhibition

Mutant	Mutated gene	Protein	Insertion site (n.t.)^*a*^	Function of the mutated gene
M60 (Δ*ctaA*)	*ctaA*	Heme A synthase	1023	ATP synthesis pathway
M47 (Δ*hemeY*)	*hemeY*	Protoporphyrinogen oxidase	773	ATP synthesis pathway
YU1 (Δ*qoxA*)	*qoxA*	Quinol oxidase subunit II	751	ATP synthesis pathway
YU2 (Δ*guaB*)	*guaB*	IMP dehydrogenase	462	GTP synthesis pathway

^*a*^ Transposon insertion site; nucleotide (n.t.) number from translation initiation codon.

### Deficiency in energy generation increases the susceptibility of *S. aureus* to lugdunin

**Fig 2:**
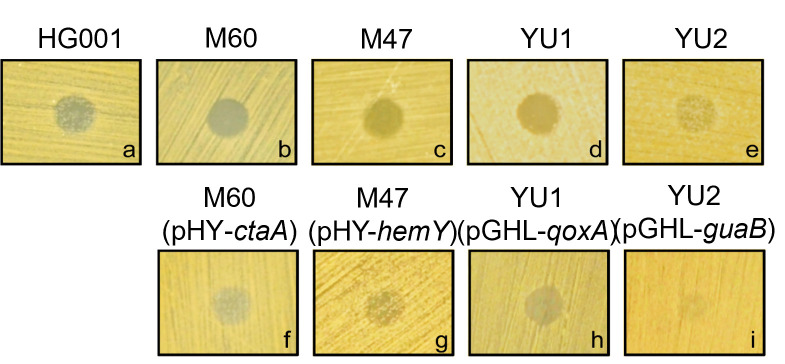
Susceptibility of energy-generation deficient mutants to synthetic lugdunin. Overnight culture of *S. aureus* HG001, M60, M60 (pHY*-ctaA*), M47, M47 (pHY-*hemY*), YU1, YU1 (pGHL-*qoxA*), YU2, and YU2 (pGHL-*guaB*) was spread on the agar plate. Two microliters of synthetic lugdunin (1 mg/mL) was then applied to the medium. After 48 h of incubation at 37°C, the inhibition zones in the lugdunin-containing regions were observed and photographed. The displayed image is representative of three independent experiments.

Previous studies have shown that lugdunin, secreted by *S. lugdunensis,* effectively inhibits the growth of *S. aureus* (7). This study used synthetic lugdunin to investigate whether defects in the energy-generation system affect *S. aureus* susceptibility to *S. lugdunensis* inhibition and whether this effect is specifically attributed to lugdunin secretion. To evaluate this, synthetic lugdunin was applied to plates that had been spread with *S. aureus*. After incubation, bacterial growth was examined in the presence of the synthetic lugdunin. The results showed that in regions where synthetic lugdunin was applied, the growth of *S. aureus* HG001 appeared indistinct and blurred, indicating that bacterial growth was effectively inhibited (Fig. 2a). In contrast, the energy generation-deficient mutants M60, M47, YU1, and YU2 exhibited more distinct and clearer zones in the regions containing synthetic lugdunin compared to the parental strain *S. aureus* HG001 (Fig. 2b through e). The increased susceptibility was reversed when the mutated genes were complemented (Fig. 2f through i). These findings suggest that defects in energy generation increase *S. aureus* susceptibility to lugdunin.

### *S. aureus* SCVs are more susceptible to *S. lugdunensis* and lugdunin

**Fig 3:**
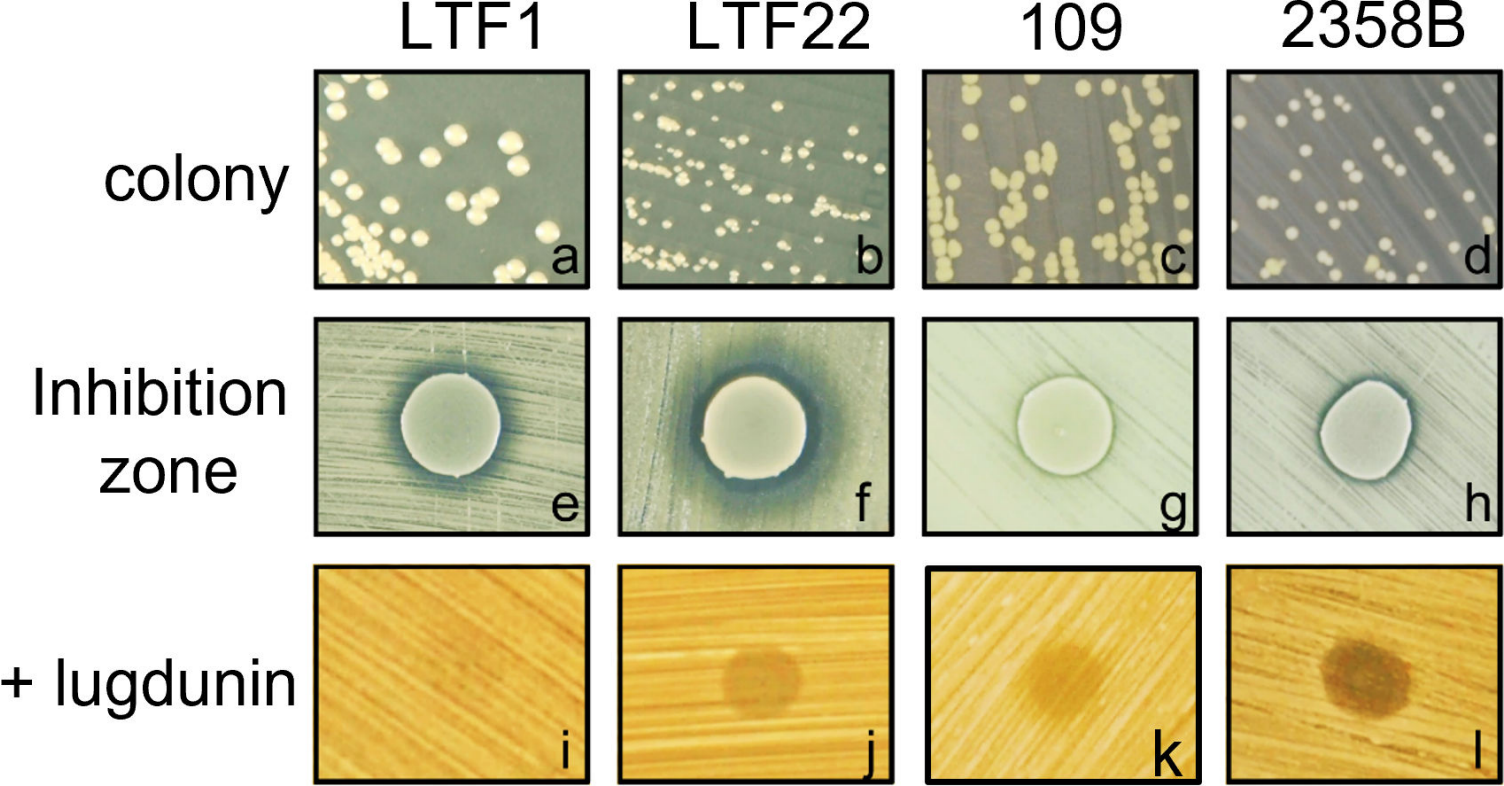
Susceptibility of *S. aureus* clinical isolates and their SCV derivatives to *S. lugdunensis* inhibition and synthetic lugdunin. Colony morphology of *S. aureus* LTF1 (**a**), its SCV derivative LTF22 (**b**), 109 (**c**), and its SCV derivative 2358B (**d**). Inhibition zones surrounding *S. lugdunensis* (**e–h**) on 2-DP containing BM medium and in lugdunin-containing regions (**i–l**) were observed and photographed. The displayed image is representative of three independent experiments.

*S. aureus* SCVs are recognized as a significant clinical challenge due to their strong association with persistent and relapsing infections (4). SCVs often emerge from mutations that affect the electron transport chain and energy metabolism (23). The M60, M47, YU1, and YU2 mutants exhibit SCV-like characteristics and show increased susceptibility to *S. lugdunensis* and lugdunin (Fig. 2). To further assess the susceptibility of clinically isolated SCVs to *S. lugdunensis* and lugdunin, we analyzed clinical MRSA strains (LTF1 and 109) and their SCV derivatives (LTF22 and 2358B) (15, 16). As shown in Fig. 3, the SCV derivatives LTF22 and 2358B exhibited smaller colony sizes compared to their respective parental strains LTF1 and 109 (Fig. 3a through d). Inhibition assays revealed that the SCV derivatives LTF22 and 2358B were more susceptible to *S. lugdunensis* inhibition compared to their parental strain LTF1 and 109 (Fig. 3e through h). A similar result was observed when synthetic lugdunin was applied (Fig. 3i through l). The results indicate that clinical SCVs are more susceptible to *S. lugdunensis* inhibition and lugdunin, supporting the hypothesis that metabolic deficiency sensitizes *S. aureus* to lugdunin in a clinically meaningful context.

### Lugdunin increases the susceptibility of SCVs to antibiotics

**Fig 4:**
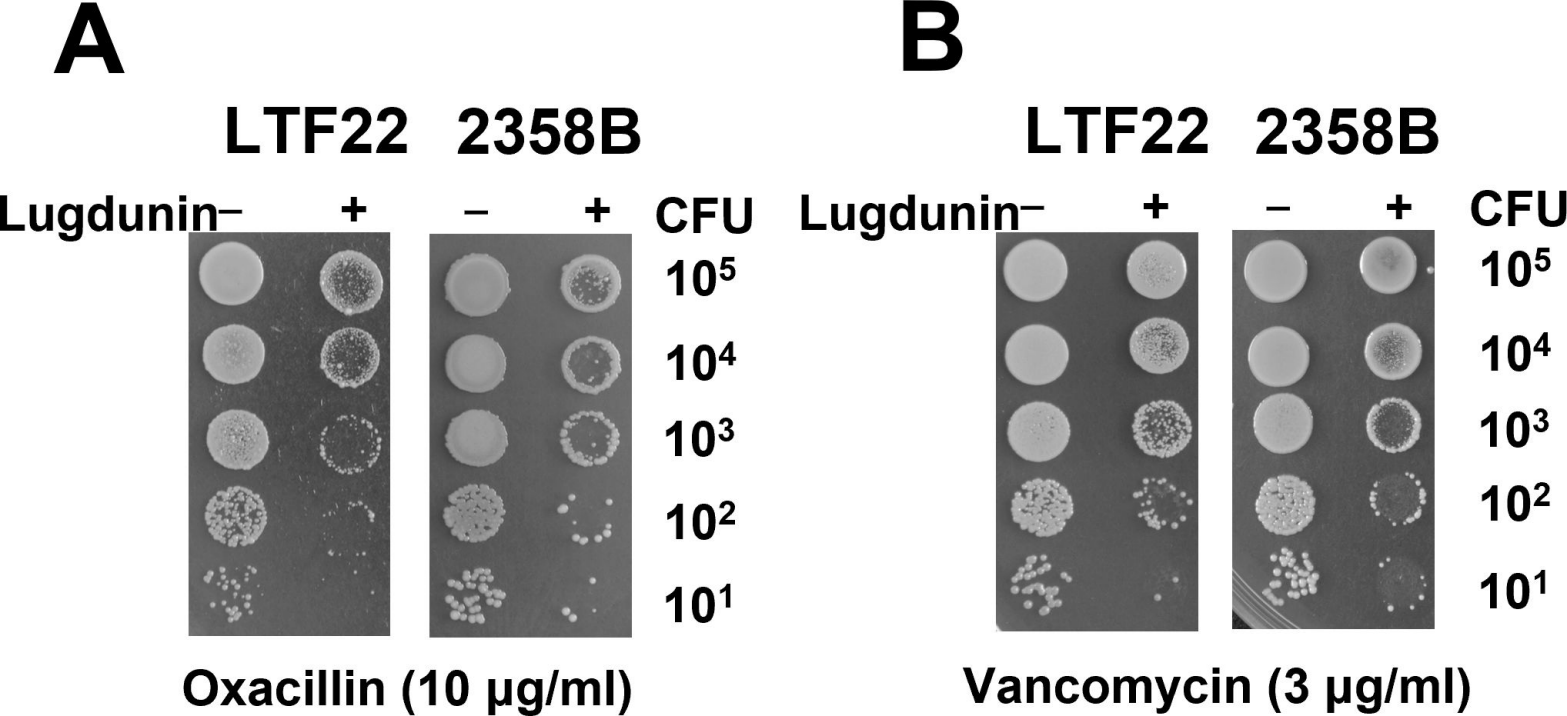
Effect of lugdunin on drug susceptibility of *S. aureus* clinical SCV strains LTF22 and 2358B. Cells were serially diluted and 10 µL of each dilution was mixed with or without lugdunin and then spotted on plates containing (A) oxacillin (10 µg/mL) or (B) vancomycin (3 µg/mL). The plates were incubated for 24 h and subsequently photographed. The bacterial inoculum ranged from 10^5^ to 10^1^ colony-forming units (CFUs) for both strains. The displayed image is representative of three independent experiments.

To determine whether lugdunin increases the susceptibility of SCVs to antibiotics, a spot assay was performed. The spot assay is a rapid, sensitive, and reliable method for detecting low-level antibiotic resistance without the limitation of log2 dilutions, which are typically used in antimicrobial susceptibility tests. The results showed that in the absence of lugdunin, *S. aureus* SCV strains LTF22 and 2358B exhibited substantial growth on plates containing oxacillin (10 µg/mL) and vancomycin (3 µg/mL). However, when treated with lugdunin, these SCV strains displayed significantly increased susceptibility to both oxacillin and vancomycin (Fig. 4), indicating that lugdunin enhances the inhibitory effects of these antibiotics. The results suggest that lugdunin sensitizes *S. aureus* SCV strains to oxacillin and vancomycin, effectively overcoming their resistance and enhancing antibiotic efficacy.

## DISCUSSION

Bacterial energy metabolism plays a fundamental role in cellular homeostasis, survival, and resistance to environmental stresses, including antibiotics and antimicrobial peptides (24, 25). In this study, we identified four genes, *ctaA*, *hemY*, *qoxA*, and *guaB,* which play crucial roles in ATP and GTP synthesis (14, 21, 22). Disruption of these genes significantly increased the susceptibility of *S. aureus* to *S. lugdunens*is and lugdunin. The mutants lacking these genes exhibited SCV phenotypes, which are characterized by slow growth, reduced metabolism, and increased antibiotic tolerance (4). These findings align with previous studies showing that defects in heme biosynthesis and electron transport chain components contribute to SCV formation and alter bacterial responses to antimicrobial agents (26–28). The strong correlation between metabolic deficiencies and enhanced susceptibility to *S. lugdunens*is and lugdunin emphasizes that a potential therapeutic approach involves targeting bacterial energy metabolism to increase the effectiveness of antimicrobial compounds. To clarify whether the increased susceptibility of the identified mutants could be attributed to growth defects, we monitored the growth curves of *S. aureus* HG001 and the mutant strains. As shown in Fig. S3, all mutants exhibited significantly slower growth compared to the HG001 strain, consistent with their metabolic defects. To ensure that the observed susceptibility to *S. lugdunensis* is attributable to antimicrobial activity rather than growth differences, we adjusted the initial bacterial densities prior to lawn preparation. Specifically, the energy-deficient mutants were plated at a higher optical density than their parental strain, HG001, to compensate for slower growth. As a result, all strains, including mutants, achieved full confluence on agar surfaces within the same incubation period. This adjustment minimized the impact of growth kinetics on inhibition zone measurements and allowed for a more accurate assessment of *S. lugdunensis* inhibitory effects. Moreover, similar trends were observed in assays using synthetic lugdunin applied directly to the bacterial lawns (Fig. 2), further confirming that the increased inhibition observed in mutants is due to their intrinsic susceptibility to lugdunin, rather than growth-related effects. While we cannot fully exclude the influence of growth rate, our findings strongly implicate metabolic deficiencies as a key factor contributing to the enhanced susceptibility phenotype. The consistent susceptibility pattern observed in both energy-deficient mutants and clinical SCVs further supports the conclusion that metabolic impairment plays a central role in sensitizing *S. aureus* to lugdunin.

Lugdunin’s potent activity against metabolically compromised *S. aureus* suggests that ATP depletion and metabolic stress exacerbate bacterial susceptibility to antimicrobial peptides. ATP is essential for numerous cellular functions, including efflux pump activity, membrane maintenance, and repair mechanisms (24). Inhibiting ATP production can, therefore, make bacteria more susceptible to antimicrobial stressors, reducing their ability to mount an effective defense. This raises intriguing questions regarding the broader applicability of metabolic-targeted therapies in treating drug-resistant bacterial infections. If bacterial ATP synthesis pathways can be selectively inhibited, they may serve as a novel target for adjuvant therapies that enhance the efficacy of existing antibiotics, particularly against resilient SCV populations.

SCVs are frequently associated with chronic and relapsing infections, such as osteomyelitis and prosthetic joint infections, and pose significant clinical challenges due to their ability to persist in host tissues, evade immune responses, and exhibit increased resistance to commonly used antibiotics (29, 30). SCVs arise due to mutations in metabolic pathways, particularly those affecting the electron transport chain, ATP production, and heme biosynthesis (30, 31). In addition to laboratory strains, we validated these findings using clinical MRSA strains (109 and LTF1) and their SCV derivatives (LTF22 and 2358B). Our study demonstrated that clinical SCVs (LTF22 and 2358B) were significantly more susceptible to *S. lugdunensis* and synthetic lugdunin than their respective parental strains (LTF1 and 109). This observation suggests that SCVs, due to their already compromised metabolic state, may be particularly vulnerable to antimicrobials that target cellular energy pathways. One possible reason for this increased susceptibility lies in the mechanism of lugdunin, which acts as a cation ionophore (11), disrupting membrane potential and leading to rapidly exhausting cellular energy reserves. To further determine whether the increased susceptibility observed in energy-deficient mutants is specific to lugdunin or extends to other membrane-active agents, we tested the protonophore carbonyl cyanide m-chlorophenyl hydrazone (CCCP). Similar to lugdunin, CCCP disrupts the membrane potential by dissipating the proton motive force (32). Our results showed that both HG001-derived energy-deficient mutants and clinical SCVs exhibited enhanced sensitivity to CCCP compared to their respective parental strains (Fig. S1). These findings suggest that defects in energy metabolism sensitize *S. aureus* to membrane depolarizing agents in general, likely due to the additive energetic burden imposed by such compounds. This supports the hypothesis that lugdunin’s protonophore-like action has amplified effects in energy-compromised cells and highlights the role of metabolic state in determining susceptibility to ionophores and related membrane-disruptive agents. SCVs, which already suffer from impaired ATP synthesis, may be unable to compensate for the additional metabolic burden imposed by lugdunin, resulting in heightened sensitivity. These findings support the notion that lugdunin could serve as an effective therapeutic option for SCV-associated infections, which are difficult to treat due to their resistance to conventional antibiotics. Although we performed MIC assays using synthetic lugdunin (data not shown), no difference in MIC values was observed between the wild-type strain and energy-deficient mutant strains. This result suggests that MIC determinations alone may not adequately reflect the functional susceptibility differences related to bacterial energy metabolism. A similar situation has been extensively documented in vancomycin-intermediate *S. aureus* (VISA) and heterogeneous VISA (hVISA) strains, which often display MIC values comparable to those of vancomycin-susceptible *S. aureus*, yet exhibit markedly reduced responsiveness to treatment in clinical settings (33). Likewise, in our study, the energy-compromised mutants and clinical SCVs, despite sharing similar MICs with their parental strains, demonstrated significantly increased susceptibility to lugdunin in spot inhibition assays. These discrepancies highlight the limitations of conventional MIC testing in capturing physiologically relevant tolerance phenotypes.

Previous studies have shown that nasal colonization with *S. lugdunensis* correlates with a reduced prevalence of *S. aureus* in the same niche (7). This raises an interesting avenue for further research, specifically exploring whether *S. lugdunensis* could be harnessed as a probiotic or a natural microbial competitor to prevent *S. aureus* infections. Given the strain-dependent heterogeneity in lugdunin production among *S. lugdunensis* isolates (34), we evaluated several clinical strains prior to this study. Among these, strain L10 showed the strongest and most consistent inhibitory activity against *S. aureus* in our assays and was therefore selected for further analysis. To validate that the observed inhibitory effects were attributable to lugdunin rather than other strain-specific factors, we also employed synthetic lugdunin in parallel experiments. The similar susceptibility profiles observed with both the L10 strain and synthetic lugdunin support the conclusion that lugdunin activity, rather than other components, underlies the observed effects.

One of the most promising findings in this study is the ability of lugdunin to enhance SCV susceptibility to conventional antibiotics. Due to their slow metabolic rate and altered cell wall structure, SCVs typically exhibit reduced susceptibility to β-lactams, aminoglycosides, and glycopeptides (28, 35). Our results indicate that treatment with lugdunin significantly increased SCV susceptibility to oxacillin and vancomycin, suggesting a synergistic effect between the antimicrobial peptide and these antibiotics. Lugdunin has been shown to form water-filled channels and alter bacterial membrane permeability (12). This structural alteration may enhance the uptake of oxacillin and vancomycin by facilitating their diffusion through the bacterial cell envelope, ultimately increasing their efficacy against *S. aureus* SCVs. Another potential mechanism underlying this synergy is that lugdunin-induced membrane depolarization triggers bacterial cell envelope stress responses, leading to an increased turnover of peptidoglycan precursors. This process may force SCVs into a more metabolically active state, rendering them more susceptible to cell wall-targeting antibiotics. A similar phenomenon has been observed with other antimicrobial peptides, such as daptomycin, which disrupts bacterial membrane integrity and increases the effectiveness of β-lactams against MRSA (36, 37). Our findings raise the possibility that lugdunin could be incorporated into existing treatment regimens to overcome antibiotic resistance in persistent *S. aureus* infections. In addition to its potential as an antimicrobial agent, lugdunin holds promise for clinical application in two key areas: nasal decolonization and combination therapy. Its natural role in eliminating *S. aureus* from the human nasal microbiota suggests that topical formulations of lugdunin, or its synthetic derivatives, may be effective in preventing colonization and reducing the risk of subsequent invasive infections. Moreover, our data demonstrate that lugdunin significantly enhances the efficacy of oxacillin and vancomycin against SCVs, highlighting its potential as an adjuvant agent in co-therapy. Given its membrane-targeting activity and minimal propensity for resistance development, synthetic lugdunin analogs with improved stability and pharmacokinetics may offer a novel strategy for treating persistent, antibiotic-resistant staphylococcal infections. Additionally, structural modifications of lugdunin to enhance its stability and bioavailability have been further explored, potentially improving its clinical utility (10). Notably, *S. aureus* has shown minimal potential for developing resistance to lugdunin, which may be due to its multifaceted mechanism of action (11, 12, 38). This stands in stark contrast to conventional antibiotics, where resistance frequently emerges through target modification, efflux pump activation, or enzymatic degradation. Given the increasing prevalence of antibiotic-resistant bacteria, the development of combination therapies that enhance the effectiveness of existing antibiotics is of paramount importance.

Our findings provide strong evidence that lugdunin has significant therapeutic potential, particularly for treating persistent *S. aureus* infections. Its ability to sensitize SCVs to antibiotics suggests that it could serve as an adjuvant therapy, enhancing the efficacy of existing treatments. Moreover, its potent activity against metabolically compromised bacteria indicates its potential to target antibiotic-resistant strains. As antibiotic resistance continues to rise globally, innovative approaches, such as targeting bacterial metabolism with lugdunin, may offer new avenues for combating drug-resistant bacterial infections.

## SUPPLEMENTAL MATERIAL

Supplemental material spectrum.01006-25-s0001.pdf Fig. S1 to S3; Supplemental methods.

ASM does not own the copyrights to Supplemental Material that may be linked to, or accessed through, an article. The authors have granted ASM a non-exclusive, world-wide license to publish the Supplemental Material files. Please contact the corresponding author directly for reuse.
