# Supplementary material for: Defects in energy metabolism increase the susceptibility of Staphylococcus aureus and its small colony variants (SCVs) to Staphylococcus lugdunensis and lugdunin
Source: Microbiol Spectr. 2025 Aug 22;13(10):e01006-25. doi: 10.1128/spectrum.01006-25 (PMC12502701; doi:10.1128/spectrum.01006-25)
Supplement: Supplemental material — Fig. S1 to S3; Supplemental methods. [file spectrum.01006-25-s0001.pdf]

## Supplementary methods

### Total RNA extraction and quantitative RT-PCR

Bacterial cells were treated with lysostaphin (0.5 mg/mL; Sigma-Aldrich) and incubated at 37 °C for 15 minutes to facilitate cell lysis. Total RNA was then extracted and purified using TRIzol reagent (Invitrogen, Waltham, MA, USA) in accordance with the manufacturer's protocol. RNA transcript levels were quantified by real-time reverse transcription PCR (RT-PCR) using a Bio-Rad system. Target genes, including *ctaA*, *hemY*, *qoxA*, and *guaBc* were reverse transcribed and amplified using the following specific primer pairs: CtaA-F (5'- GCA GCTCCGATTAATGCTTG-3') and CtaA-R (5'- CATGGTGCG TTG ATT CCA GA-3'; hemY-F (5'- GCTACA ATA CGT CAG TGG AT-3') and hemY-R (5'- AATGCT GGA TCT TGTCCG AA-3'); qoxA-F (5'- CACCCA TTTGTC GTAGTCTT-3') and qoxA-R (5'- GATTCCACA ATTAGGTGGTC-3'); guaB-F (5'- AGCAGAAGCATTAATGGGTA-3') and guaB-R (5'- TGT ATTCACTGGAGCTGTAA-3'). The housekeeping gene *gyrB* was used as an internal control and amplified using primers gyrB-F1 (5′-ACGGATAACGGACGTGGTATCCCA) and gyrB-R1 (5′-GCCACCGCCGAATTTACCACCA). To verify amplification specificity, melting-curve analysis was performed following RT-PCR. Each experiment was conducted independently at least three times, with all samples analyzed in triplicate.

### Growth curve analysis

Overnight cultures of *S. aureus* HG001, M60, M47, YU1, and YU2 were diluted to an initial optical density at 578 nm ($OD_{578}$) of 0.1 in fresh TSB. The cultures were incubated at 37°C with shaking, and $OD_{578}$ readings were measured hourly over a 24-hour period.

## Supplementary figures

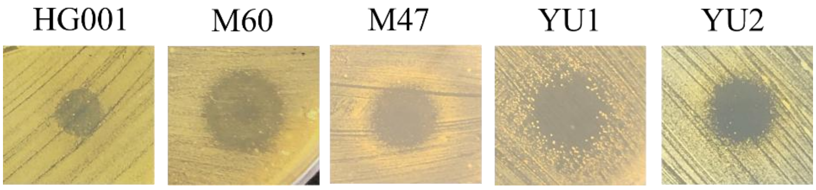


**Figure S1. Susceptibility of energy-deficient mutants to the CCCP.** Overnight cultures of *S. aureus* HG001, M60, M47, YU1, and YU2 were spread on agar plates, and 2 µl of CCCP (1 mg/ml) was then applied to the medium. Images are representative of three independent experiments.

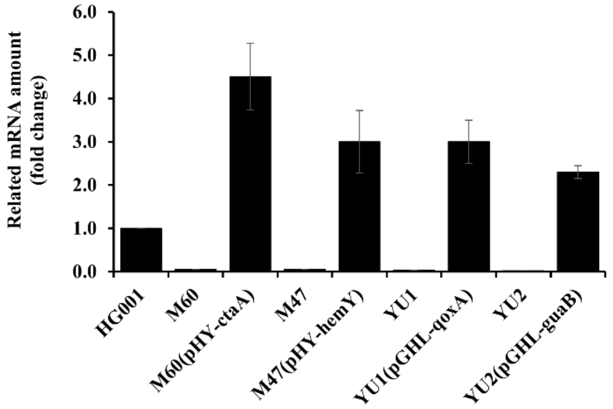


**Figure S2. RT-qPCR analysis of gene expression in *S. aureus* HG001, M60, M60(pHY-*ctaA*), M47, M47(pHY-*hemY*), YU1, YU1(pGHL-*qoxA*), YU2 and YU2(pGHL-*guaB*).** Total RNA was isolated and relative expression levels of *ctaA*, *hemY*, *qoxA*, and *guaB* were measured and normalized to *gyr* RNA. The RNA amounts of *ctaA*, *hemY*, *qoxA*, and *guaB* in *S. aureus* HG001 were set at 1 to calculate the relative amounts of *ctaA*, *hemY*, *qoxA*, and *guaB* in mutant strains and complemented strains. Data represent mean ± SD from three independent experiments.

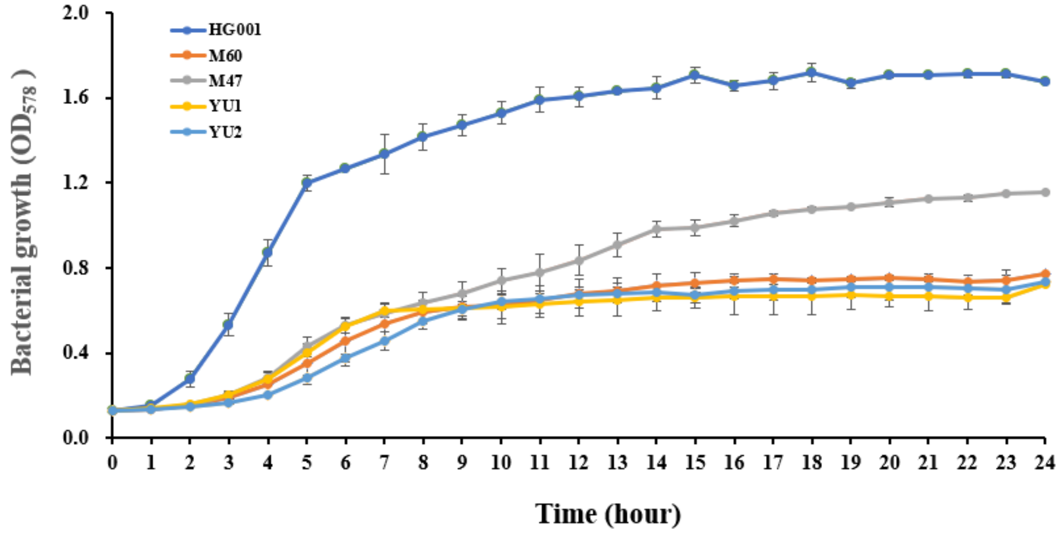


**Figure S3. Growth curves of *S. aureus* HG001, M60, M47, YU1 and YU2.** Overnight cultures were diluted into fresh TSB and incubated at 37°C with shaking. $OD_{578}$ was measured hourly for 24 hours. Data represent mean ± SD from three independent experiments.
